# Identification of Immune Activation Markers in the Early Onset of COVID-19 Infection

**DOI:** 10.3389/fcimb.2021.651484

**Published:** 2021-09-03

**Authors:** Johannes J. Kovarik, Anna K. Kämpf, Fabian Gasser, Anna N. Herdina, Monika Breuer, Christopher C. Kaltenecker, Markus Wahrmann, Susanne Haindl, Florian Mayer, Ludwig Traby, Veronique Touzeau-Roemer, Katharina Grabmeier-Pfistershammer, Manuel Kussmann, Oliver Robak, Harald Willschke, Care Ay, Marcus D. Säemann, Klaus G. Schmetterer, Robert Strassl

**Affiliations:** ^1^Department of Internal Medicine III, Division of Nephrology and Dialysis Medical University Vienna, Vienna, Austria; ^2^Department of Laboratory Medicine, Institute of Clinical Virology, Medical University of Vienna, Vienna, Austria; ^3^Department of Pathology, Medical University Vienna, Vienna, Austria; ^4^Department of Laboratory Medicine, Medical University of Vienna, Vienna, Austria; ^5^Department of Internal Medicine I, Medical University of Vienna, Vienna, Austria; ^6^Department of Dermatology, Medical University Vienna, Vienna, Austria; ^7^Institute of Immunology, Center for Pathophysiology, Infectiology and Immunology, Medical University Vienna, Vienna, Austria; ^8^Department of Anaesthesia, Intensive Care Medicine and Pain Medicine, Medical University of Vienna, Vienna, Austria; ^9^6th Medical Department With Nephrology and Dialysis, Wilhelminen Hospital, Vienna, Austria; ^10^Sigmund Freud University, Vienna, Austria

**Keywords:** COVID-19, SARS-CoV-2, cytokines, inflammatory markers, infection

## Abstract

This study aimed to determine the specific cytokine profile in peripheral blood during the early onset of COVID-19 infection. This was a cross-sectional exploratory, single center study. A total of 55 plasma samples were studied. Serum samples of adults showing symptoms of COVID-19 infection who were tested positive for SARS-CoV-2 infection (CoV+, n=18) at the COVID-19 outpatient clinic of the Medical University of Vienna were screened for immune activation markers by Luminex technology. Additionally, age and gender-matched serum samples of patients displaying COVID-19 associated symptoms, but tested negative for SARS-CoV-2 (CoV-, n=16) as well as healthy controls (HC, n=21) were analyzed. COVID-19 positive (CoV+) patients showed a specific upregulation of BLC (141; 74-189 pg/mL), SCD30 (273; 207-576 pg/mL), MCP-2 (18; 12-30 pg/mL) and IP-10 (37; 23-96 pg/mL), compared to patients with COVID19-like symptoms but negative PCR test (CoV-), BLC (61; 22-100 pg/mL), sCD30L (161; 120-210 pg/mL), MCP-2 (8; 5-12 pg/mL) and IP-10 (9; 6-12 pg/mL) and healthy controls (HC) (BLC 22; 11-36 pg/mL, sCD30 74; 39-108 pg/mL, MCP-2 6; 3-9. pg/mL, IP-10 = 8; 5-13). The markers APRIL, sIL-2R, IL7, MIF, MIP-1b, SCF, SDF-1a, sTNF-RII were elevated in both CoV+ and CoV- patient groups compared to healthy controls. HGF, MDC and VEGF-A were elevated in CoV- but not CoV+ compared to healthy controls. BLC, sCD30, MCP-2 and IP-10 are specifically induced during early stages of COVID-19 infection and might constitute attractive targets for early diagnosis and treatment of this disease.

## Introduction

According to World Health Organization (WHO), the outbreak of a novel coronavirus strain termed severe acute respiratory syndrome coronavirus type 2, SARS-CoV-2 at the end of the year 2019 has led to an unprecedented global pandemic having infected an estimated more than 80 million people. First case descriptions have focused on the respiratory symptoms of the disease such as cough, shortness of breath and, in severe cases, low oxygenation requiring hospitalization and ventilation (Lescure et al., 2020). Furthermore, SARS-CoV-2 infection induces pathologies in multiple organs which contributes to the unusually high lethality (Becher and Frerichs, 2020). It is now agreed that one of the most severe clinical problems is a state of hyperinflammation induced by SARS-CoV-2 infection during early stages of the disease (de la Rica et al., 2020; Reddy et al., 2020). Inflammation is governed by the interaction of multiple different cell types such as granulocytes, macrophages, endothelial along with epithelial cells which interact with cells of the adaptive immune system. Many of these interactions are regulated by the early secretion of cytokines, chemokines, and other messenger substances resulting in an exacerbation of the inflammatory reaction, that can culminate in a systemic cytokine storm also activating inflammasomes affecting multiple compartments of the organism (Rodrigues et al., 2021). Accordingly, several studies have aimed to better define the inflammatory pattern induced by Coronavirus Disease (COVID)-19 infection. First reports in January 2020 from a Chinese cohort described high serum levels of inflammatory cytokines with the chemokine CXCL10/IP-10 showing the most pronounced increase and allowing stratification of risk patients (Huang et al., 2020) which was also corroborated in an Israelian cohort (Lev et al., 2021). Similarly, a more recent study analyzing 185 serum markers of inflammation and cardiovascular disease has identified several significantly up-regulated inflammatory markers in the peripheral blood of hospitalized SARS-CoV-2 patients (Sims et al., 2020). A broad up-regulation of inflammatory cytokines and chemokines has also been described in Chinese cohorts from the first wave as well (Chi et al., 2020; Zhao et al., 2020). Subsequently, numerous studies have further addressed COVID-19 induced hyperinflammation drawing a multi-faceted picture with different immunological processes contributing to immediate and late pathologies in SARS-CoV-2 infection (Buszko et al., 2021). Treatment strategies concomitantly focused on this cytokine storm and treatment options investigating approved anti-inflammatory agents such as the interleukin-(IL)-6 blocker tocilizumab (Guaraldi et al., 2020) and the IL-1 blocker anakinra have been explored (Cavalli et al., 2021). Most notably, recent trials using the broadly anti-inflammatory glucocorticoids showed promising results regarding mortality in critically ill patients (Horby et al., 2020). Surprisingly, though it has not been established yet whether the overall quality or quantity of the SARS-CoV-2 induced cytokine inflammatory response differs from that caused by other common respiratory infections. Better understanding of the underlying mechanisms of specific individuals, which may alter the propensity of individual patients to experience over exuberant inflammatory responses after viral infection is urgently needed (Forbester and Humphreys, 2020). Furthermore, a deeper knowledge of disease specific cytokine patterns especially at the early onset of the disease is necessary to specifically ameliorate clinical symptoms and to personalize treatment.

A detailed characterization of the cytokine pattern during the early onset of the COVID-19 infection in comparison to healthy controls and symptomatic but COVID negative persons is lacking so far. Hence in this study peripheral blood specimens from symptomatic patients at the COVID outpatient clinic of the Medical University of Vienna were stratified into patients tested COVID-19 negative and positive with age- and sex-matched healthy volunteers as control group.

## Methods

### Ethics Statement

This exploratory study is in compliance with the Helsinki Declaration (Ethical Principles for Medical Research Involving Human Subjects) and was conducted in accordance with the guidelines of research boards at the study site. This single-center study was approved by the local ethics committee of the Medical University of Vienna (EC#1280/2020).

### Patient Recruitment and Detection of SARS-CoV-2 by RT-PCR

Healthy volunteers were recruited at the Vienna General Hospital (AKH Wien) Medical University of Vienna. COVID-19 symptomatic patients were randomly included by order of appearance at the COVID-19 outpatient ward of the Medical University of Vienna. Onset of symptoms was anamnestically determined and for this study only patients with a symptom onset ≤2 days were included. At the time of presentation a serum blood draw was performed and samples were stored at -80°C and similarly respiratory swabs were taken once from the nasopharyngeal cavity of each patient to determine the SARS-CoV-2 status. RT-PCR was performed on the fully-automated Roche Cobas 6800 RT-PCR system (Roche Diagnostics GmbH, Rotkreuz, Switzerland) from respiratory swab samples agitated in 0,9% saline solution (NaCl). The cobas^®^ SARS-CoV-2 assay was used according to the manufacturer’s instructions for the detection of SARS-CoV-2 specific ribonucleic acid (RNA). According to clinical routine testing protocols, all analyses were performed within six hours after sample extraction. The control group was recruited from age- and sex-matched healthy volunteers from the General Hospital of Vienna between 2017 and the end of 2019. As this was prior to the COVID-19 pandemic, SARS-CoV-2 PCR was not performed in this group.

### Analysis of Inflammatory Markers in Patient Serum

Undiluted blood serum samples were analyzed using the ProcartaPlex™ Multiplex Immunoassay (Human immune monitoring 65 Plex, Thermo Fisher Scientific, Reference Number MAN0017980) according to manufacturer’s instruction. Of the 65 analytes, 39 were below the lower limit of quantification in >95% of all samples (irrespective of group) and were therefore excluded from further analysis. Measurements and analysis of all Human ProcartaPlex Immunoassays were performed on a Luminex 200 instrument (Luminex Corp., Austin, Tx, USA) as described previously (Muhlbacher et al., 2020). In short, patient samples were adjusted to 10 mM EDTA to prevent the complement-mediated prozone effect. Then, 50 µL of the Magnetic Bead solution was added to each well of the provided 96-well flate bottom plate. After washing of magnetic beads, 25 µL of Universal Assay Buffer (1x) was added to each well, followed by 25 uL of patient sample, prepared standards or blank [Universal Assay Buffer (1x)]. The plate was incubated at RT in the dark on a plate shaker at 550 rpm for 60 min. After incubation, the plate was washed and incubated with pre-mixed Detection Antibody Mixture (25 µL/well) as above for 30 min. Subsequently, the beads were washed to remove unbound antibody, 50 µL of Streptavidin-PE (SAPE) solution was added to each well and the incubation step was repeated. As a final step, 120 µL of Reading Buffer was added to each well after washing and plates were processed on a Luminex 200 instrument.

### Statistical Analysis

Each analyte was plotted separated by patient group to assess data distribution. Group differences between the three cohorts were tested using Kruskal-Wallis-test in conjunction with Dunn’s multiple comparisons test using multiplicity adjusted calculation of p-values. Correlation analyses were calculated using a Spearman correlation algorithm. GraphPad Prism 6.05 (GraphPad Software, CA, USA) was used for statistical analysis and data presentation.

## Results

### Patient Recruitment and Characteristics

A total of 34 patients who presented to the COVID-19 outpatient ward of the Medical University of Vienna between March 2020 and August 2020 with typical COVID-19 symptoms including cough, fever, sore throat, myalgia, loss of taste and/or smell, fatigue or dyspnoea were selected from the database for this cross-sectional analysis. Patients with anamnestic symptom onset within the last two days before presentation were included into the study. All patients were tested for the presence of SARS-CoV-2 RNA by routine PCR testing from nasopharyngeal swabs at the time of presentation and stratified into a symptomatic COVID-19 negative (CoV-; n=16) and a symptomatic COVID-19 positive (CoV+; n=18) group (PCR Ct-value: median 24.7 [interquartile range IQR 13.2-41.1]. At this time point also a venous blood draw for inflammatory marker determination was performed. 21 age and sex matched healthy volunteers were recruited and served as control group. Characteristics from all three groups are shown in **Table 1**.

**Table 1:** Patient characteristics of healthy control (HC) volunteers, symptomatic, COVID-19 negative tested patients (CoV-) and symptomatic, COVID-19 positive tested patients (CoV+).

	HC (n = 21)	CoV- (n = 16)	CoV+ (n = 18)
female: male (%)	5 : 16	4 : 12	3 : 15
age (y; median, range)	42.0 (20-63)	35.0 (20-74)	42.0 (21-69)
cough	/	13 (0.81)	17 (0.94)
fever	/	12 (0.75)	6 (0.33)
sore throat	/	5 (0.31)	6 (0.33)
myalgia	/	3 (0.19)	6 (0.33)
loss of taste and/or smell	/	1 (0.06)	6 (0.33)
fatigue	/	2 (0.13)	4 (0.22)
dyspnea	/	9 (0.56)	3 (0.17)
hospitalization	/	3 (0.19)	7 (0.39)

Data are given in absolute numbers and in brackets relative to the whole group (100%). For age, the total range is given in brackets. Group comparison with ANOVA showed no significant differences between gender and age between the three study groups.

### Assessment of Inflammatory Markers in Serum Specimens

Serum specimen from the three study groups were subjected to analysis using a commercially available 65 analyte multiplex immunoassay panel containing most relevant cytokines, chemokines and soluble receptors and growth factors typically found in inflammation (refer to **Supplementary Material 1** for the full list). Thereby, we aimed to analyze a broad range of factors produced by cells of the innate as well as the adaptive immune system, thus covering the breadth of the SARS-CoV-2 induced immune response. Out of the 65 assessed molecules, 39 were below the lower limit of quantification in >95% of all samples (irrespective of group) and were therefore excluded from further analysis (marked with an *asterisk in **Supplementary Material 1**). Of note, these excluded analytes also contained IFN-γ and IL-8 which were described as elevated in COVID-19 positive patients in other studies on hospitalized patients (Galani et al., 2021). Individual levels of analyzed proteins above the detection limit are shown in **Supplementary Material 2**. 10 analytes were observed at detectable levels in the sera of all three groups but showed no significant differences between the healthy control group and the specimens from either of the symptomatic groups. The respective analytes and their levels in all three groups are summarized in **Table 2**.

**Table 2:** Median serum concentration, 25% quartile and 75% quartile in pg/mL of analytes showing no significant differences in specimen from healthy control (HC) volunteers, symptomatic, COVID-19 negative tested patients (CoV-) and symptomatic, COVID-19 positive tested patients (CoV+).

Analyte (pg/mL)	HC (n = 21)	CoV-(n = 16)	CoV+(n = 18)
CD40L	9 [5 ; 118]	68 [46 ; 118]	45 [24 ; 79]
ENA-78	227 [187 ; 277]	214 [102 ; 597]	150 [72 ; 1054]
Eotaxin	37 [23 ; 43]	54 [16 ; 63]	36 [25 ; 44]
Eotaxin-2	127 [74 ; 167]	193 [96 ; 285]	179 [84 ; 409]
IL-16	64 [33 ; 150]	13 [13 ; 127]	45 [13 ; 356]
IL-17A	32 [23 ; 83]	90 [23 ; 166]	32 [23 ; 401]
MCP-1	83 [41 ; 117]	129 [29 ; 173]	126 [74 ; 269]
MIP-1a	5 [2 ; 19]	8 [5 ; 22]	9 [3 ; 14]
MMP-1	20 [11 ; 48]	29 [5 ; 66]	63 [17 ; 134]
TWEAK	1133 [842 ; 1509]	1470 [1185 ; 2139]	1186 [918 ; 1652]

### COVID-19 Infection Presents With a Distinct Inflammatory Pattern Compared to Other Common Respiratory Infections

**Figure 1:**
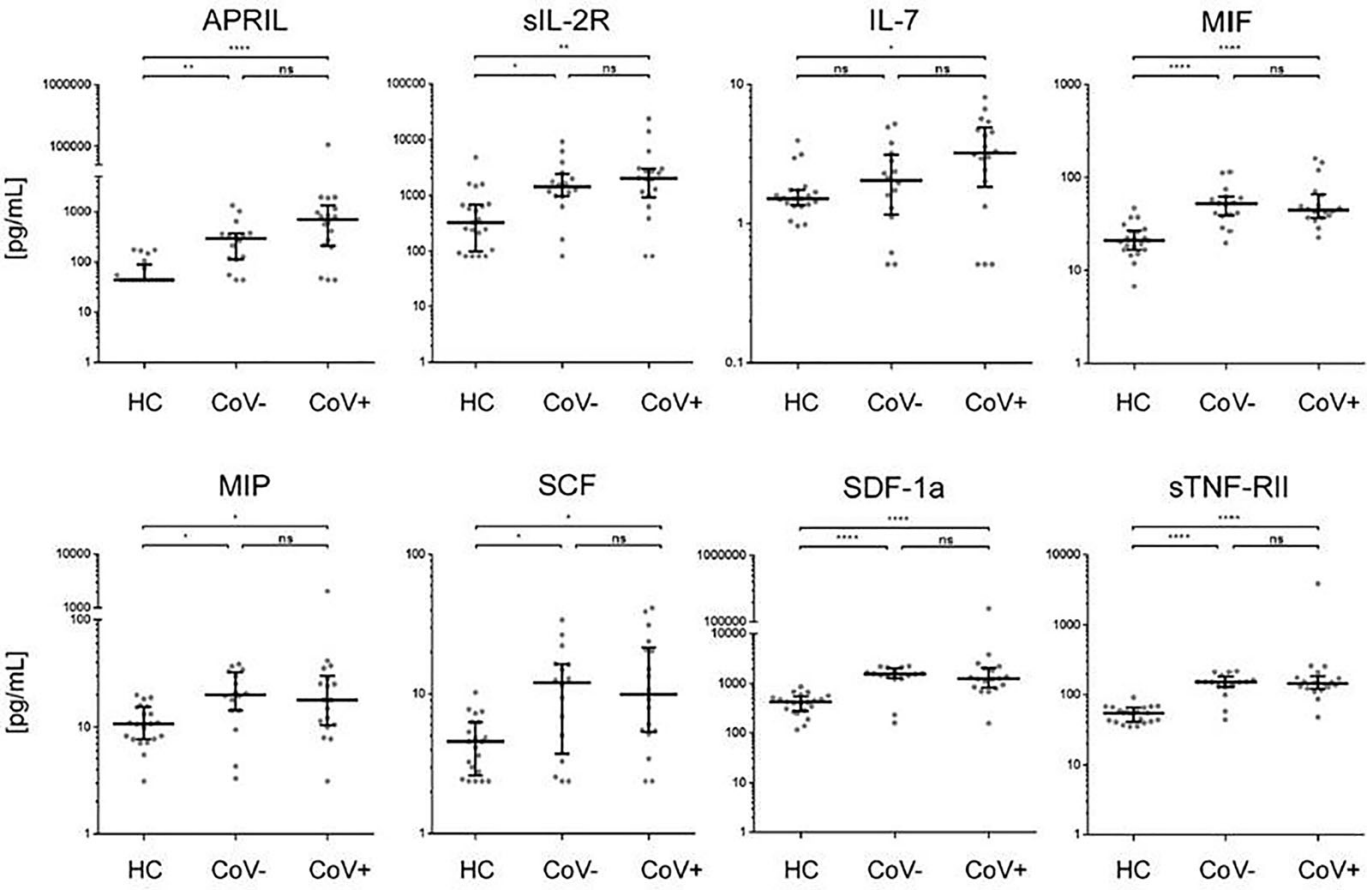
Serum markers elevated in COVID-19- and COVID-19+ symptomatic patients compared to healthy controls. The indicated inflammatory markers were assessed by multiplex analysis from the serum of healthy control (HC) volunteers, symptomatic, COVID-19 negative tested patients (CoV-) and symptomatic, COVID-19 positive tested patients (CoV+). For each marker, individual results are depicted as dot plots. Each point represents the median of two technical replicates of each individual. Data are shown on a logarithmic scale for better visualization. Data are shown as median and interquartile range 25%-75%. p > 0.05 = ns not significant; *p < 0.05, **p < 0.01, ****p < 0.0001, Group differences between the three cohorts were tested using Kruskal-Wallis-test in conjunction with Dunn’s multiple comparisons test using multiplicity adjusted calculation of p-values. (HC, n=21), (CoV-, n=16), (CoV+, n=18); APRIL, a proliferation-inducing ligand; sIL-2R-soluble interleukin 2 receptor; IL-7, interleukin 7; MIF, Macrophage migration inhibitory factor; MIP, macrophage inflammatory proteins; SCF, stem cell factor; SDF, 1a-stromal cell-derived factor 1; sTNF, RII-tumor necrosis factor receptor.

**Figure 2:**
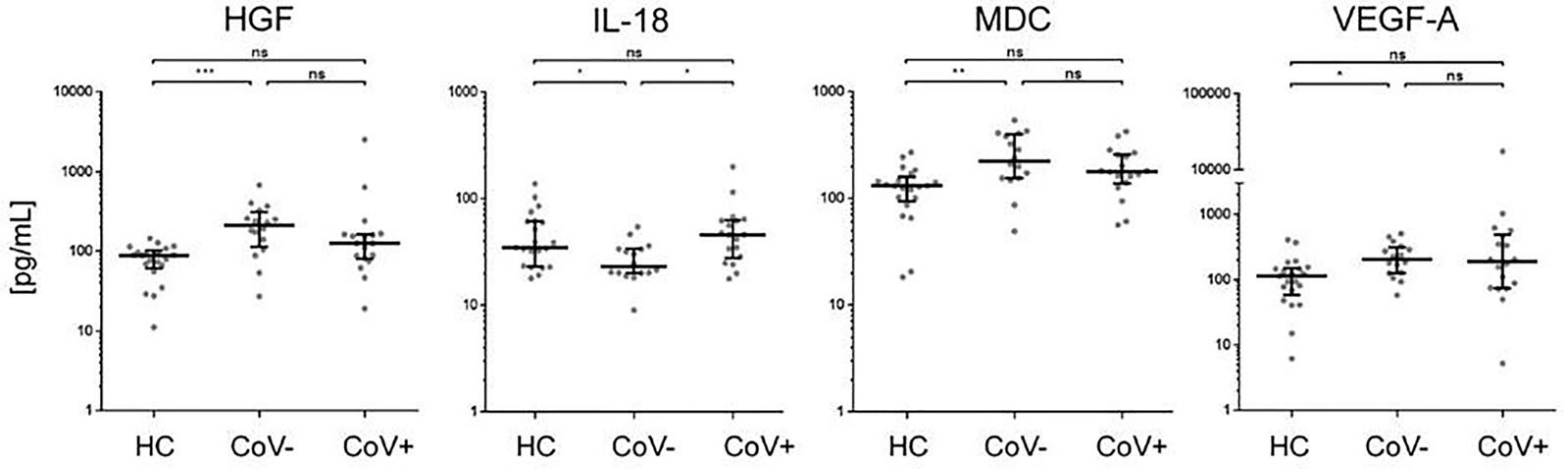
Serum markers elevated or downregulated markers in COVID-19- patients compared to healthy controls and COVID-19+ patients. The indicated inflammatory markers were assessed by multiplex analysis from the serum of healthy control (HC) volunteers, symptomatic, COVID-19 negative tested patients (CoV-) and symptomatic, COVID-19 positive tested patients (CoV+). For each marker, individual results are depicted as dot plots. Each point represents the median of two technical replicates of each individual. Data are shown on a logarithmic scale for better visualization. Data are shown as median and interquartile range 25%-75%. p > 0.05 = ns not significant; *p < 0.05, **p < 0.01, ***p < 0.001, Group differences between the three cohorts were tested using Kruskal-Wallis-test in conjunction with Dunn’s multiple comparisons test using multiplicity adjusted calculation of p-values. (HC, n=21), (CoV-, n=16), (CoV+, n=18). (HC, n=21)); HGF, hepatocyte growth factor; IL-18, interleukin-18; MDC, macrophage-derived chemokine; VEGF-A, vascular endothelial growth factor A.

In accordance with a general inflammatory response in respiratory infections, 7 analytes (APRIL, sIL-2R, MIF, MIP-1b, SCF, SDF-1a, sTNF-RII) showed a significant increase in serum levels in both the CoV- and CoV+ group compared to the healthy control cohort (**Figure 1** and **Supplementary Material 3**), while remaining statistically indifferent between CoV- and CoV+. These markers include the soluble form of TNFSF13 (tumor necrosis factor ligand superfamily member 13), also known as APRIL (A proliferation-inducing ligand), which nevertheless showed a clear trend towards higher serum levels in the CoV+ compared to the CoV- group (median; IQR25%-75% HC: CoV-: CoV+ = 44; 44-89 pg/mL: 290; 112-370 pg/mL: 598; 207-1055 pg/mL). All other markers showed a robust increase compared to the control cohort but no apparent differences between the CoV- and CoV+ symptomatic group. Thus, these proteins can be considered as general markers of respiratory inflammation. Additionally, IL-7 was significantly up-regulated in the CoV+ but not in the CoV- group compared to the control group while showing no significant difference between the two symptomatic groups. In this respect, IL-7 levels were generally very low with all sera analyzed showing values <10 pg/mL (**Figure 1** and **Supplementary Material 3**). Of note, four further analytes were significantly up-regulated in the symptomatic CoV- group but not in the CoV+ group. HGF (median, IQR25%-75% HC: CoV-: CoV+ = 89; 61-102 pg/mL: 212; 114-310 pg/mL: 126; 80-164 pg/mL), MDC (median, IQR25%-75% HC: CoV-: CoV+ = 131; 94-159 pg/mL: 224; 155-399 pg/mL: 178; 137-257 pg/mL) and VEGF-A (median, IQR25%-75% HC: CoV-: CoV+ = 115; 58-151 pg/mL: 207; 126-315 pg/mL: 189; 74-494 pg/mL) were significantly up-regulated in the symptomatic COV- group while the CoV+ group showed no significant difference compared to the control group (**Figure 2** and **Supplementary Material 3**). VEGF-A levels showed a high variance within the CoV+ group ranging from near baseline values to extremely high concentrations (total range: 5-17545 pg/mL) suggesting that this protein might be very heterogeneously affected by COVID-19 infection. Intriguingly, IL-18 was significantly down-regulated in the symptomatic CoV- group but remained at healthy control levels in the CoV+ group (median, IQR25%-75% HC: CoV-: CoV+ = 34; 23-61 pg/mL: 23; 20-33 pg/mL: 45; 27-63 pg/mL).

**Figure 3:**
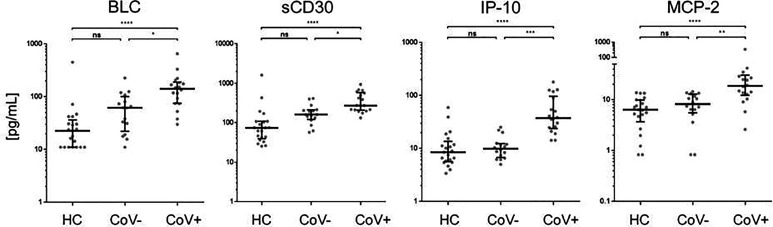
Immune activation markers specifically elevated in the serum of COVID-19+ patients compared to COVID- patients and healthy controls. The indicated inflammatory markers were assessed by multiplex analysis from the serum of healthy control (HC) volunteers, symptomatic, COVID-19 negative tested patients (CoV-) and symptomatic, COVID-19 positive tested patients (CoV+). For each marker, individual results are depicted as dot plots. Each point represents the median of two technical replicates of each individual. Data are shown on a logarithmic scale for better visualization. Data are shown as median and interquartile range 25%-75%. p > 0.05 = ns not significant; *p < 0.05, **p < 0.01, ***p < 0.001, ****p < 0.0001, Group differences between the three cohorts were tested using Kruskal-Wallis-test in conjunction with Dunn’s multiple comparisons test using multiplicity adjusted calculation of p-values. (HC, n=21), (CoV-, n=16), (CoV+, n=18); BLC, chemokine B lymphocyte chemoattractant; sCD30, soluble CD30; IP-10, interferon-gamma induced protein 10; MCP-2, monocyte chemotactic protein-2.

Of note, four analytes showed no differences between the healthy control and the symptomatic CoV- group but were significantly up-regulated in the CoV+ group. These were BCL/CXCL13, soluble (s)CD30, IP-10 and MCP-2/CCL8 (**Figure 3** and **Supplementary Material 3**). BLC (median, IQR25%-75% HC: CoV-: CoV+ = 22; 11-36 pg/mL: 61; 22-100 pg/mL: 141; 74-189 pg/mL) and sCD30 (median, IQR25%-75% HC: CoV-: CoV+ = 74; 39-108 pg/mL: 161; 120-210 pg/mL: 273; 207-576 pg/mL) also showed a trend towards higher serum levels in symptomatic CoV- patients but were even further increased in the CoV+ group. For IP-10 (median, IQR25%-75% HC: CoV-: CoV+ = 8; 5-13 pg/mL: 10; 7-12 pg/mL: 37; 24-97 pg/mL) and MCP-2 (median, IQR25%-75% HC: CoV-: CoV+ = 6; 3-10 pg/mL: 8; 5-12 pg/mL: 18; 12-30 pg/mL) no differences between the healthy control and the symptomatic CoV- group were observable. Correlation analyses of these four parameters did not reveal robust correlation in any of the analyzed groups underscoring a highly individual inflammatory response in symptomatic SARS-CoV-2 negative and positive patients (**Supplementary Material 4**). Taken together, our analyses thus identify a unique COVID-19 specific inflammatory signature of four serum proteins in an early onset of disease setting.

## Discussion

By performing Luminex based Multiplex Immunoassay analysis in negative and positive tested cohorts with clinical signs of COVID-19 infection, we were able to identify an exclusive upregulation of BLC, sCD30, MCP-2 and IP-10 in patients early after onset of SARS-CoV-2 infection.

It has been shown previously that patients with severe COVID-19 infection display a hyperinflammatory milieu in the circulation with concomitant elevation of a variety of inflammatory cytokines and vascular endothelial damage markers and it has been now extensively discussed that the SARS-Cov-2 induced “cytokine storm” is a major source of complications but also provides promising therapeutic targets (Sinha et al., 2020; Chen and Quach, 2021; Mulchandani et al., 2021). Interestingly though, coronavirus infections trigger a very distinct immunological program, which is especially seen by the lack or delayed onset of a type I interferon response (Blanco-Melo et al., 2020; Hadjadj et al., 2020).

Dampening the excessive inflammation induced by SARS-CoV-2 infection has been the focus of clinical management. Accordingly, recent data on the ratio of the pro-inflammatory cytokine IL-6 and the anti-inflammatory cytokine IL-10 suggested that serum cytokines may be used for risk stratification in hospitalized COVID-19 patients (McElvaney et al., 2020). These observations were based on increased levels of multiple cytokines most prominently IL-6. Yet, the data were obtained from critically ill patients potentially also suffering from multiple co-morbidities (Tang et al., 2020).

Our study dissected the systemic cytokine response in symptomatic COVID-19 patients at the time of first hospital contact at the COVID outpatient clinic and compared results of COVID-positive and -negative tested individuals to each other and to healthy controls. We focused on the early inflammatory response of the COVID-19 infection and not on aged, hospitalized and often polymorbid patients.

In line with our results, it has been shown previously, that IP-10, a marker that induces T-cells and is therefore important for antiviral defense, is upregulated in hospitalized COVID-19 positive patients (Huang et al., 2020). Serum concentrations of IP-10 were higher in ICU patients compared to non-ICU patients. It has been shown that serum IP-10 levels are highly associated with disease severity and predict the progression of COVID-19 (Yang et al., 2020; Lev et al., 2021). Typically, IP-10 is associated as downstream chemokine of an IFN-γ response, although it can also be induced by other stimuli such as LPS or stressors such as heat shock or UV light *in vitro* [reviewed in (Vazirinejad et al., 2014)]. Along those lines, it is now discussed whether IFN-γ and IP-10 levels are independently affected by SARS-CoV-2 (Buszko et al., 2021). In contrast to previous studies, IFN-γ serum levels in all our cohorts were below the detection limit. This could be due to our recruitment strategy focusing on patients early after onset of symptoms. Thus, it could be hypothesized that an early IP-10 response in SARS-CoV-2 infection is followed by a later induction of IFN-γ which in turn further enhances IP-10 production. In this regard, it will be interesting to observe the results of the ongoing trial with the IFN-γ blocking antibody Emapalumab (trial NCT04324021) and how this affects the inflammatory situation in COVID-19 patients and the overall clinical outcome.

In our study, patients with severe COVID-19 infection display high levels of the monocyte chemoattractant MCP-2 (Sims et al., 2020), a chemokine that utilizes multiple cellular receptors to attract and activate different immune cells (Proost et al., 1996). Transcriptional analyses of respiratory cell populations and serum analysis in response to SARS-CoV-2 infection show strong upregulation of MCP-2 (Blanco-Melo et al., 2020; Zizzo and Cohen, 2020).

Therefore, anti-CCL8/MCP-2 antibodies might offer a novel point of intervention in the management of patients with COVID-19 induced hyperinflammation. Yet, our analyses also identified a significant upregulation of sCD30, a marker implicated in T-cell response and BLC/CXCL13 in CoV+ compared to symptomatic yet CoV- patients. sCD30 is a member of the tumor-necrosis-factor receptor superfamily. Low serum levels of sCD30 were found in healthy humans, whereas increased sCD30 concentrations were measured in autoimmune diseases such as systemic lupus erythematosus, rheumatoid arthritis, certain viral infections and adult T cell leukemia/lymphoma (van der Weyden et al., 2017).

A limited number of studies assessed the physiological role of CD30/CD30 ligand interactions in control of infection and it has been shown that after Epstein-Barr (EBV) infection, large numbers of CD30+ cells are generated (Zhou et al., 2017). However, the regulation of this factor early after COVID-19 infection has not been elucidated in detail so far.

BLC/CXCL13 on the other hand, is a small homeostatic CXC family chemokine that is highly expressed in the pleural cavity and critically involved in B-cell recruitment during infection (Ansel et al., 2002). A recent study has identified BLC as novel biomarker for lethal SARS-Cov-2 infection (Horspool et al., 2021). The authors, also found a correlation between BLC levels and antibody production. From these observations and the known biological role, it might be deduced that BLC is crucial in mediating the adaptive immune response e.g. in follicular B helper cells after COVID-19 infection and promotes antibody production. Interestingly, contrary, to previously published results in critically ill patients (Guaraldi et al., 2020), we could not detect IL-6, IL-8 and IFN-γ in our tested cohorts. However, in accordance with a previous study, an explanation might be the very early onset of infection at the time of blood withdrawal in our analysis (Zhang et al., 2020). Of note, correlation analyses of the four COVID-19 specific parameters only revealed low correlations between individuals. This indicates that patients show highly individual inflammatory responses to COVID-19 infection. Therefore, for potential diagnostic or therapeutic settings, all four parameters have to be considered in parallel.

We further demonstrated an upregulation of the chemokines APRIL, sIL-2R, IL-7, MIF, MIP-1b, SCF, SDF-1a, sTNF-RII in CoV+ patients, which was not specific for SARS-CoV-2 infection but also found in the COV- group. Still, these findings support the general finding that SARS-CoV-2 induces a broad cytokine storm. Several of these markers have been associated with distinct features of SARS-CoV-2 infection. Recently, a potential contribution of increased soluble IL-2R to lymphopenia in COVID-19 patients has been indicated (Zhang et al., 2020). IL-7 is a pleiotropic cytokine (Laterre et al., 2020), currently studied in clinical trials for oncologic and infectious disorders (Mackall et al., 2011). An upregulation of this cytokine early after COVID-19 infection might be an important immunomodulatory step of the host immunity in response to the infection.

MIF is an important regulator of innate immunity (Calandra and Roger, 2003). Previous studies showed decreased MIF levels and selective modulation after coronavirus infection (Frieman et al., 2008). MIP serum levels were found to be elevated in patients with COVID-19, requiring ICU admission (Huang et al., 2020). Similarly, serum SCF concentrations were significantly higher in fatal than severe or mild COVID-19 cases (Xu et al., 2020). sTNF-RII inhibit the TNF-alpha biological effects and thus may constitute a negative regulatory mechanism in acute inflammation. However, we did not find a significant upregulation of IL-18, a cytokine participating in host defense against infection, in early COVID-19 infection. This discrepancy might be due to different expression kinetics in the course of SARS-CoV-2 infection. In accordance to previous studies which indicate severe dysregulation of type I interferon responses in COVID-19, where these central mediators of the immune response seem actually to be blunted, we did not measure any interferon levels in our analysis (Acharya et al., 2020; Meffre and Iwasaki, 2020).

Some limitations should be addressed for our analysis. First, clinical data were only available from one center. Due to the exploratory study design, the number of analysed patients is relatively small and should be confirmed by larger longitudinal multi-center trials. Second, no detailed follow up of the investigated subpopulations could be provided within the scope of this study, hence, the viral genesis of disease of CoV- persons could not be ultimately confirmed. In this respect, especially a stratification of symptomatic SARS-CoV-2 negative patients according to the underlying pathogen (e.g. other endemic human coronaviruses, influenza A/B, etc.) would further add to the understanding of respiratory viral infections. Third the clinical assessment of COVID-19 symptoms was subjective according to the clinician at the COVID-19 triage at the outpatient clinic. Fourth, although restricted to a maximum of two days, the individual duration of disease symptoms varied between the persons before appearance at the outpatient clinic.

In conclusion, our study demonstrates that BLC/CXCL13, sCD30, MCP-2/CCL8 and IP-10/CXCL10 are specifically and individually upregulated in symptomatic COVID-19-positive patients but not in patients suffering from other respiratory infections with similar symptoms. Thus, our study might help to gain novel insights into the specific systemic immune response of the host, early after COVID-19 infection and ultimately might help to develop innovative therapeutic strategies against COVID-19 infection.

## Data Availability Statement

The raw data supporting the conclusions of this article will be made available by the authors, without undue reservation.

## Ethics Statement

The studies involving human participants were reviewed and approved by The ethics committee of the Medical University of Vienna (EC#1280/2020). Written informed consent for participation was not required for this study in accordance with the national legislation and the institutional requirements.

## Author Contributions

JK, KS, and RS conceived the study and wrote the first draft. AH and MB helped with sample processing. AK, SH, and MW carried out the Luminex analysis. CK and FG provided support for statistical analysis. FM, LT, VT-R, KG-P, and MK provided support in recruiting and analysis of the study populations. MS read and edited the draft. All authors contributed to the article and approved the submitted version.

## Funding

The study was funded by the Medical Scientific Fund of the Mayor of the City of Vienna (project#COVID010).

## Conflict of Interest

The authors declare that the research was conducted in the absence of any commercial or financial relationships that could be construed as a potential conflict of interest.

## Publisher’s Note

All claims expressed in this article are solely those of the authors and do not necessarily represent those of their affiliated organizations, or those of the publisher, the editors and the reviewers. Any product that may be evaluated in this article, or claim that may be made by its manufacturer, is not guaranteed or endorsed by the publisher.

## Supplementary Material

Supplementary Material 1 Full list of 65 analytes. Analytes marked with an asterisk were below the limit of quantification.

Supplementary Material 2 Levels of analyzed proteins from individual donors/patients from the three groups.

Supplementary Material 3 Median serum concentration, 25% quartile and 75% quartile in pg/mL of analytes showing differential levels in specimen from at least one group of healthy control (HC) volunteers, symptomatic, COVID-19 negative tested patients (CoV-) and symptomatic, COVID-19 positive tested patients (CoV+).

Supplementary Material 4 Heat map of correlation of BLC, sCD30, IP-10 and MCP-2 in the three study groups. Healthy control (HC) volunteers, symptomatic, COVID-19 negative tested patients (CoV-) and symptomatic, COVID-19 positive tested patients (CoV+). Numbers indicate the respective Spearman r correlation coefficient.
