# Supplementary material for: Identification of Immune Activation Markers in the Early Onset of COVID-19 Infection
Source: Front Cell Infect Microbiol. 2021 Sep 3;11:651484. doi: 10.3389/fcimb.2021.651484 (PMC8446609; doi:10.3389/fcimb.2021.651484)
Supplement: Supplementary Material 1 — Full list of 65 analytes. Analytes marked with an asterisk were below the limit of quantification. [file DataSheet_1.docx]

APRIL - BAFF* - BLC - sCD30 - CD40L - ENA‑78 – Eotaxin - Eotaxin‑2 - Eotaxin‑3* - FGF‑2* - Fractalkine* - G‑CSF* - GM‑CSF* - GRO‑a* - HGF - IFN‑a* - IFN‑g* - IL‑1 a* - IL‑1 b* - IL‑10* - IL‑12p70* - IL‑13* - IL‑15* - IL‑16 - IL‑17A - IL‑18 - IL‑2* - IL‑20* - IL‑21* - IL‑22* - IL‑23* - IL‑27* - sIL‑2R - IL‑3* - IL‑31* - IL‑4* - IL‑5* - IL‑6* - IL‑7 - IL‑8* - IL‑9* - IP‑10 - I‑TAC* - LIF* - MCP‑1 - MCP‑2 - MCP‑3* - M‑CSF* - MDC – MIF - MIG* - MIP‑1 a - MIP‑1 b - MIP‑3 a* - MMP‑1 - NGF b* - SCF - SDF‑1 a - TNF a* - TNF b* - sTNF‑RII - TRAIL* - TSLP* - TWEAK - VEGF‑A
