# Supplementary material for: Identification of Immune Activation Markers in the Early Onset of COVID-19 Infection
Source: Front Cell Infect Microbiol. 2021 Sep 3;11:651484. doi: 10.3389/fcimb.2021.651484 (PMC8446609; doi:10.3389/fcimb.2021.651484)
Supplement: Supplementary Material 2 — Levels of analyzed proteins from individual donors/patients from the three groups. [file DataSheet_2.pdf]

Group	Mean analyte concentration (pg/mL)																								
	MCP-2	IL-2R	MIP-1 a	SDF-1 a	IP-10	IL-7	BLC	Eotaxin-2	Eotaxin	IL-17A	SCF	HGF	MIP-1 b	MCP-1	MIF	MMP-1	IL-18	IL-16	CD40L	VEGF-A	ENA-78	sCD30	TNF-RII	MDC	APRIL	TWEAK
Control	1,2	527,7	157,5	407,4	18,8	1,5	71,4	67,1	23,1	23,3	2,4	73,7	10,7	8,9	27,9	7,9	38,8	35,3	687,6	115,1	163,8	170,2	55,5	128,5	175,9	8664,7
Control	10,0	637,0	7,3	409,6	10,7	1,4	17,9	127,6	42,2	65,8	2,4	114,4	19,9	83,5	26,1	36,1	32,8	197,9	30,6	6,2	191,1	193,7	54,8	196,2	104,7	974,9
Control	7,6	81,4	2,6	309,6	8,5	1,4	11,0	66,6	41,5	23,3	2,4	90,2	13,2	115,7	18,6	28,7	22,5	35,1	4,7	41,1	214,7	62,1	35,6	132,1	44,1	844,2
Control	10,9	324,1	20,0	857,0	9,0	1,5	46,5	18,8	23,5	54,4	2,4	116,2	18,3	51,5	37,4	182,9	62,2	175,2	135,3	193,7	106,8	107,9	66,4	145,0	54,5	1350,6
Control	6,4	582,2	3,7	478,8	10,1	4,0	28,9	301,8	27,9	23,3	2,8	145,8	11,0	71,6	31,2	113,1	22,7	271,3	7,1	94,2	213,7	26,3	57,0	183,9	44,1	1242,6
Control	7,1	1472,0	35,6	513,3	4,0	3,2	450,3	168,2	28,2	141,4	4,8	90,1	7,7	26,4	22,5	15,0	34,1	65,0	252,9	372,5	387,5	74,4	43,9	147,6	173,4	2062,6
Control	4,7	4820,3	11,8	285,4	11,2	1,8	23,7	93,7	41,5	99,4	3,0	79,8	9,9	71,4	22,5	44,0	59,9	242,8	81,2	81,7	194,3	1618,2	92,1	124,2	44,1	1384,0
Control	13,8	246,1	30,3	429,0	39,1	1,6	22,6	80,7	49,5	79,5	7,8	108,7	18,9	251,9	18,3	54,3	85,6	105,1	260,7	185,0	278,7	90,5	65,4	100,9	44,1	1673,8
Control	6,2	81,4	120,0	422,6	5,4	1,3	11,0	105,3	38,0	62,5	3,6	35,0	10,9	51,4	11,9	15,0	75,3	13,9	1092,9	127,7	253,5	25,4	35,2	20,7	44,1	4256,0
Control	2,0	81,4	2,4	474,4	20,7	1,0	42,0	146,2	56,1	23,3	5,4	76,9	3,1	216,7	6,8	4,7	33,8	91,3	4,7	15,2	228,4	74,8	54,5	87,3	44,1	699,2
Control	5,1	253,0	2,4	116,3	3,4	1,3	11,0	44,8	23,6	29,9	6,3	89,3	7,7	124,8	15,2	12,6	51,8	38,0	4,7	146,2	270,9	75,1	40,8	68,4	44,1	567,4
Control	5,6	1616,6	10,8	678,7	6,6	1,4	24,3	134,6	45,1	23,3	6,3	97,1	10,6	33,8	21,1	28,9	138,2	125,1	73,0	69,5	141,0	108,8	59,5	141,6	44,1	1149,5
Control	0,8	696,7	4,6	345,2	5,8	1,8	30,3	97,5	22,2	26,6	4,1	93,5	7,1	20,5	26,3	20,5	61,2	31,5	25,3	93,9	195,2	61,0	43,4	245,0	44,1	980,1
Control	13,4	1597,3	5,4	265,9	59,2	3,0	42,0	210,2	32,6	23,3	2,4	55,9	15,5	46,0	14,6	53,1	103,7	43,8	4,7	133,9	178,8	429,9	67,9	270,5	44,1	1073,8
Control	2,7	237,9	19,6	663,4	6,6	1,6	11,0	177,0	41,0	23,3	4,9	128,6	8,2	96,7	47,3	11,8	34,9	274,5	101,5	411,7	183,6	46,4	68,9	131,4	147,9	1473,2
Control	0,8	81,4	2,4	188,9	7,5	1,0	11,0	151,4	21,6	23,3	3,3	69,5	13,2	94,2	16,8	4,7	19,3	13,5	4,7	40,9	227,7	29,2	39,7	65,8	44,1	624,8
Control	7,1	92,2	2,4	139,7	5,5	1,5	16,2	192,9	47,5	97,8	10,3	66,6	5,5	106,6	16,7	18,8	32,6	30,2	4,7	48,3	419,7	103,9	41,6	18,4	44,1	1087,4
Control	8,5	664,7	2,5	446,3	4,6	1,0	11,0	56,4	31,0	32,3	7,5	29,2	15,9	119,9	37,3	74,8	22,9	105,1	4,7	123,5	270,1	42,4	57,3	121,6	44,1	839,4
Control	5,3	105,2	2,4	587,0	6,1	1,7	30,7	96,0	19,0	34,8	4,6	11,2	8,2	36,3	21,9	31,0	17,9	58,7	4,7	156,0	275,8	33,0	44,9	171,3	44,1	1133,9
Control	13,5	372,4	2,4	247,3	11,8	1,7	11,0	166,4	37,9	88,3	4,6	27,8	7,1	106,9	19,3	10,4	39,1	13,5	4,7	144,8	395,9	36,5	36,7	102,1	168,3	682,8
Control	9,8	212,9	4,6	565,2	15,3	1,5	14,4	135,5	57,9	118,4	7,3	94,5	15,3	156,7	20,5	9,9	23,4	103,8	9,9	78,5	448,8	43,4	68,0	135,2	74,8	1545,2
COV+	18,2	1965,2	14,2	1337,7	14,2	3,6	152,4	152,1	31,6	1303,9	41,4	163,2	41,6	70,4	64,8	273,8	62,5	306,0	117,5	567,6	9080,8	658,1	131,8	176,3	1863,7	1817,7
COV+	27,1	621,1	10,0	946,7	38,5	2,4	117,8	84,9	35,9	23,3	6,1	126,6	14,9	314,2	43,5	64,9	46,2	463,9	80,6	75,3	131,5	274,2	138,4	141,9	47,6	1515,0
COV+	19,3	1964,8	3,1	1226,8	40,8	3,1	52,5	262,9	40,1	23,3	2,4	82,7	7,8	164,2	28,6	82,8	115,8	47,6	39,1	205,7	65,0	385,3	153,9	267,7	44,1	1369,3
COV+	2,6	3041,6	11,8	1224,2	35,4	3,0	134,2	207,0	24,7	23,3	5,2	75,3	10,0	102,6	39,4	105,1	17,8	189,8	64,2	204,2	111,6	613,4	117,0	201,8	272,9	1147,1
COV+	42,2	383,7	80,9	2511,9	179,6	3,3	115,9	84,8	42,5	373,0	20,3	149,2	35,4	12,2	145,6	62,0	24,9	13,5	659,3	349,8	900,6	216,0	209,2	254,1	961,0	2682,3
COV+	348,3	1021,2	12,8	787,3	118,4	4,6	168,2	117,6	50,3	589,8	31,3	88,2	37,5	337,3	50,2	19,0	20,0	13,5	15,9	336,9	2351,8	197,3	111,5	167,9	832,8	824,3
COV+	25,0	3002,0	29,2	689,5	25,8	2,9	204,4	404,7	52,4	487,5	20,8	155,1	25,4	65,1	36,8	491,9	67,9	13,5	114,4	73,6	1516,4	421,9	123,8	284,3	802,7	2113,1
COV+	35,1	1151,6	3,8	3804,8	128,4	0,5	149,2	24,8	65,2	23,3	15,1	62,1	12,2	747,3	160,9	5,6	48,0	1026,8	5,0	73,2	73,7	192,2	175,9	162,6	560,4	680,1
COV+	14,9	14019,2	17,1	1271,6	53,8	8,1	332,1	424,3	33,5	65,0	13,4	241,9	24,1	102,0	49,1	208,3	28,7	320,4	79,1	622,5	233,0	934,6	148,2	125,9	1947,3	1106,4
COV+	29,0	23901,6	15,7	158900,0	50,3	4,8	650,1	544,0	18,5	42,3	8,0	2522,7	2053,4	123,1	71,4	9,6	56,5	121,2	41,6	17545,0	169,6	273,2	3856,5	164,8	105974,8	1050,8
COV+	13,7	81,4	8,9	681,4	30,0	1,3	222,1	90,8	62,5	120,2	9,2	89,8	7,9	124,4	34,6	4,7	64,4	13,5	50,7	156,8	394,3	272,3	86,6	182,6	196,2	1211,4
COV+	24,3	2063,2	2,4	1373,2	36,3	4,3	74,8	346,6	41,1	23,3	5,4	166,7	11,4	224,0	37,1	34,5	61,7	44,0	19,5	89,1	69,5	682,4	142,0	385,3	1943,2	1161,6
COV+	12,9	81,4	2,4	157,2	21,1	0,5	29,9	30,6	11,1	23,3	2,4	19,2	3,1	3,7	22,7	4,7	34,9	13,5	4,7	5,2	100,1	132,7	47,8	56,4	44,1	497,2
COV+	14,2	2698,9	3,7	1091,3	19,1	2,0	93,1	207,4	32,5	23,3	10,8	111,6	17,8	127,6	41,8	96,1	24,2	482,0	41,5	175,1	12,5	176,1	129,2	181,2	598,9	950,2
COV+	5,8	2694,5	7,5	1984,6	89,5	0,5	175,1	17,5	7,4	23,3	3,4	46,7	10,5	254,7	120,2	20,1	200,6	837,3	46,2	50,4	38,8	492,9	260,1	95,0	415,2	332,3
COV+	10,4	2500,4	5,4	2221,9	14,3	5,7	73,6	1186,9	36,9	23,3	5,5	159,6	17,9	156,5	47,3	125,6	45,7	13,5	45,5	111,1	73,3	214,5	171,7	425,2	217,9	1866,2
COV+	9,1	6144,6	12,6	1813,0	24,7	6,7	37,8	761,3	25,2	108,7	23,9	639,7	28,2	75,4	45,9	158,1	34,1	13,5	73,1	1033,6	267,1	564,4	256,4	247,3	793,3	1596,8
COV+	54,4	1291,1	9,7	840,2	126,1	5,4	184,1	118,4	39,7	759,8	39,1	126,1	25,1	494,5	42,1	23,0	42,0	13,5	26,6	470,3	3040,8	211,5	149,6	61,0	1150,3	1259,8
COV-	9,0	1997,6	8,4	1358,1	12,6	2,8	65,8	192,0	11,9	817,6	34,0	88,6	19,4	13,7	53,5	23,7	33,9	147,1	59,2	128,6	4642,8	133,4	132,3	381,6	1330,1	1672,9
COV-	0,8	1180,3	11,8	2196,2	9,4	1,1	17,7	232,3	63,9	23,3	6,9	105,3	37,0	158,0	115,5	12,1	54,7	13,5	50,8	106,9	117,7	161,4	153,7	428,2	55,5	1164,1
COV-	0,8	1136,9	7,1	1665,4	20,1	2,1	61,9	254,0	61,0	185,3	26,6	232,3	17,9	133,4	51,4	121,2	20,2	13,5	80,5	324,6	763,6	196,4	153,0	405,5	348,5	2542,3
COV-	7,5	3934,3	24,9	2244,4	8,8	2,3	61,5	406,6	96,5	109,8	16,2	253,2	29,9	102,2	41,6	54,8	20,2	13,5	122,8	455,4	482,8	203,2	212,1	409,2	1020,0	2487,0
COV-	7,2	2575,0	8,4	1747,1	22,1	3,2	119,0	83,9	45,5	191,6	22,2	258,9	33,1	294,0	63,0	9,5	30,4	96,8	76,5	388,7	610,9	276,8	150,7	541,0	373,9	2081,8
COV-	14,5	1575,9	36,6	2099,2	7,4	1,7	73,4	186,5	15,1	123,2	9,5	141,7	34,1	105,9	112,7	69,6	33,7	13,5	231,5	126,4	28,1	213,2	150,8	286,3	212,8	1408,4
COV-	13,3	9277,0	13,4	1222,4	6,2	2,0	19,1	206,2	58,4	112,2	16,5	404,3	19,5	126,6	28,9	120,4	21,5	13,5	94,0	238,6	441,2	115,8	189,0	173,1	361,8	1488,3
COV-	3,6	162,8	5,0	234,3	5,0	0,5	11,0	74,7	21,6	23,3	2,4	27,3	3,3	38,0	26,6	4,7	9,0	137,5	28,5	58,2	25,1	62,1	44,1	49,3	44,1	572,5
COV-	13,5	1268,4	7,5	1423,5	11,5	4,9	31,1	480,8	72,0	24,9	15,0	328,3	21,1	198,8	57,7	95,7	24,8	13,5	18,2	229,6	209,5	148,1	163,1	155,2	264,2	884,0
COV-	13,7	1638,8	7,5	1464,2	10,8	3,8	107,4	653,7	56,8	109,0	12,6	239,1	25,4	174,7	41,6	37,7	18,9	13,5	60,5	275,4	558,9	398,7	153,2	209,5	317,7	2158,4
COV-	10,6	1749,4	9,8	1602,5	8,4	1,6	33,3	133,8	51,9	23,3	5,1	176,0	20,5	160,9	60,4	35,5	20,4	507,5	105,1	511,1	147,7	190,9	129,7	239,4	645,7	1452,6
COV-	5,2	987,7	2,4	1530,2	10,4	1,3	38,2	162,6	56,7	23,3	2,4	182,9	9,4	315,6	40,3	30,4	36,1	13,5	42,5	289,2	86,0	160,9	135,1	325,5	111,0	1427,0
COV-	11,7	957,2	2,7	1570,4	6,5	2,4	124,7	296,3	72,8	181,4	11,6	369,0	14,4	168,8	38,7	4,7	20,2	86,4	44,7	180,9	755,6	143,4	99,4	199,9	342,7	1618,4
COV-	6,4	81,4	112,7	159,8	6,2	0,5	15,6	57,6	14,3	46,2	2,6	53,9	4,3	3,7	19,8	4,7	18,3	341,3	871,7	172,4	210,4	57,0	58,2	86,9	117,9	6963,7
COV-	6,4	625,7	36,4	1242,7	12,0	5,2	80,0	19,3	7,6	72,2	3,3	679,0	38,5	26,6	75,4	4,7	46,4	13,5	153,0	92,9	97,5	86,5	212,8	155,0	126,6	1085,3
COV-	10,5	6167,4	5,4	2127,0	25,2	0,6	226,6	194,9	40,9	23,3	12,9	192,2	14,3	3,7	53,9	27,0	32,0	27,4	58,6	185,8	218,5	412,8	218,2	148,3	44,1	1247,3
