# Supplementary material for: Identification of Immune Activation Markers in the Early Onset of COVID-19 Infection
Source: Front Cell Infect Microbiol. 2021 Sep 3;11:651484. doi: 10.3389/fcimb.2021.651484 (PMC8446609; doi:10.3389/fcimb.2021.651484)
Supplement: Supplementary Material 3 — Median serum concentration, 25% quartile and 75% quartile in pg/mL of analytes showing differential levels in specimen from at least one group of healthy control (HC) volunteers, symptomatic, COVID-19 negative tested patients (CoV-) and symptomatic, COVID-19 positive tested patients (CoV+). [file DataSheet_3.pdf]

analyte	HC	COV-	COV+
APRIL	44.07 [44.07 ; 89.72]	290.98 [112.68 ; 370.91]	598.94 [207.06 ; 1055.65]
sIL-2R	324.1 [98.7 ; 680.7]	1422.2 [964.8 ; 2430.7]	2014.2 [921.2 ; 3011.9]
IL-7	1.5 [1.4 ; 1.8]	2.1 [1.2 ; 3.1]	3.2 [1.8 ; 4.9]
MIF	21.1 [16.8 ; 27.1]	52.5 [39.1 ; 62.4]	44.7 [37.0 ; 66.4]
MIP-1b	10.7 [7.7 ; 15.4]	20.0 [14.3 ; 32.3]	17.8 [10.4 ; 30.0]
SCF	4.6 [2.6 ; 6.3]	12.1 [3.7 ; 12.1]	10.0 [5.4 ; 21.6]
SDF-1a	422.6 [275.6 ; 539.2]	1550.3 [1271.6 ; 2011.1]	1249.2 [827.0 ; 2043.9]
sTNF-RII	54.8 [41.2 ; 65.9]	151.9 [130.3 ; 182.5]	145.1 [122.1 ; 184.2]

analyte	HC	COV-	COV+
HGF	89.3 [61.3 ; 102.9]	212.2 [114.4 ; 310.9]	126.3 [80.8 ; 164.1]
IL-18	34.9 [23.2 ; 61.7]	23.2 [20.2 ; 33.8]	45.9 [27.7 ; 63.0]
MDC	131.4 [94.1 ; 159.4]	224.4 [155.1 ; 399.5]	178.7 [137.9 ; 257.5]
VEGF-A	115.1 [58.9 ; 151.1]	207.7 [127.0 ; 315.7]	189.6 [74.8 ; 494.7]

analyte	HC	COV-	COV+
BLC	22.6 [11.0 ; 36.4]	61.7 [22.1 ; 100.5]	141.7 [74.5 ; 189.2]
sCD30	74.4 [39.4 ; 108.3]	161.2 [120.2 ; 210.7]	273.7 [207.9 ; 576.6]
IP-10	8.4 [5.6 ; 13.6]	9.9 [6.8 ; 12.4]	37.4 [23.8 ; 96.8]
MCP-2	6.4 [3.7 ; 9.9]	8.2 [5.5 ; 12.9]	18.8 [12.3 ; 30.5]
