# Supplementary figures and images for: Identification of Immune Activation Markers in the Early Onset of COVID-19 Infection
Source: Front Cell Infect Microbiol. 2021 Sep 3;11:651484. doi: 10.3389/fcimb.2021.651484 (PMC8446609; doi:10.3389/fcimb.2021.651484)

# healthy control

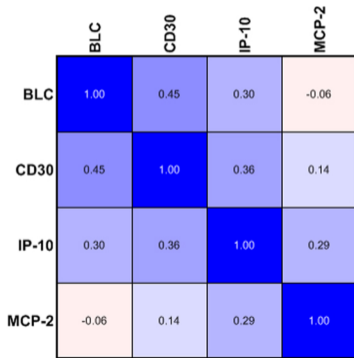

# CoV-

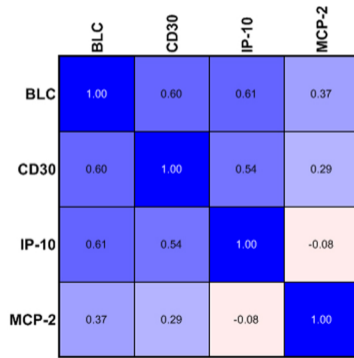

# CoV+

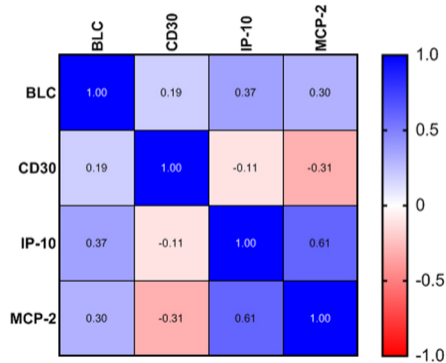

Supplement: Supplementary Material 4 — Heat map of correlation of BLC, sCD30, IP-10 and MCP-2 in the three study groups. Healthy control (HC) volunteers, symptomatic, COVID-19 negative tested patients (CoV-) and symptomatic, COVID-19 positive tested patients (CoV+). Numbers indicate the respective Spearman r correlation coefficient. [file DataSheet_4.pdf]
